# SnS_2_ and SnO_2_ Nanoparticles Obtained from Organotin(IV) Dithiocarbamate Complex and Their Photocatalytic Activities on Methylene Blue

**DOI:** 10.3390/ma13122766

**Published:** 2020-06-18

**Authors:** Jerry O. Adeyemi, Damian C. Onwudiwe

**Affiliations:** 1 Material Science Innovation and Modelling (MaSIM) Research Focus Area, Faculty of Natural and Agricultural Science, Mafikeng Campus, North-West University, Private Bag X2046, Mmabatho 2735, South Africa; jerryadeyemi1st@gmail.com; 2 Department of Chemistry, Faculty of Natural and Agricultural Science, Mafikeng Campus, North-West University, Private Bag X2046, Mmabatho 2735, South Africa

**Keywords:** dithiocarbamate, pyrolysis, nanoparticles, tin sulfide, tin oxide

## Abstract

This work reports the photocatalytic degradation of methylene blue (MB) dye using SnS_2_ and SnO_2_ nanoparticles obtained from a solvothermal decomposition (in oleylamine) and pyrolysis (in a furnace) processes, respectively, of the diphenyltin(IV) *p*-methylphenyldithiocarbamate complex. The complex, which was used as a single-source precursor and represented as **[(C_6_H_5_)_2_Sn(L)_2_]** (L = *p*-methylphenyldithiocarbamato), was synthesized and characterized using various spectroscopic techniques and elemental analysis. The structural properties and morphology of the as-synthesized nanoparticles were studied using X-ray diffraction (XRD) technique and transmission electron microscopy (TEM). UV-visible spectroscopy was used to study the optical property. The hexagonal phase of SnS_2_ and tetragonal SnO_2_ nanoparticles were identified, which exhibited varying sizes of hexagonal platelets and rod-like morphologies, respectively. The direct band gap energies of both materials, estimated from their absorption spectra, were 2.31 and 3.79 eV for SnS_2_ and SnO_2_, respectively. The photocatalytic performances of the SnS_2_ and SnO_2_ nanoparticle, studied using methylene blue (MB) as a model dye pollutant under light irradiation, showed that SnO_2_ nanoparticles exhibited a degradation efficiency of 48.33% after 120 min reaction, while the SnS_2_ nanoparticles showed an efficiency of 62.42% after the same duration of time. The higher efficiency of SnS_2_ compared to the SnO_2_ nanoparticles may be attributed to the difference in the structural properties, morphology and nature of the material’s band gap energy.

## 1. Introduction

The continuous environmental pollution by different dyes released from different human and industrial activities has stimulated the need for sustained fundamental and applied research in the area of environmental remediation [1]. Most waste water from industrial effluents contains dyes such as methylene blue (MB), rhodamine B (RhB) and methyl violet (MV) [2]. It is important to remove these dyes from water in order to ensure its reusability, since some of them are highly toxic and carcinogenic [3]. Methylene blue is a heterocyclic aromatic dye which belongs to the class of recalcitrant dyes (azo dyes) [4,5]. Its increased usage in the textile industries and potential health hazards have necessitated the need to devise a way to remove it from waste water before being reused. It causes increased heart rate, vomiting and tissue necrosis in humans [4,6]. Different physical, chemical and biological techniques have been developed for the removal of these pollutants and the alleviation of their negative impact on the environment [7]. Most of these techniques are impeded by high energy costs and incomplete degradation. For example, while the adsorption process generates secondary waste, methods such as reverse osmosis and coagulation are economically not viable and do not completely remove recalcitrant pollutants such as dyes [1,5,8,9]. Heterogeneous photocatalysis is considered as a cost-effective alternative with the potential to effect the complete decomposition of dyes from wastewater. This requires the use of semiconductor nanomaterials such as metal sulfides and metal oxides [10]. The process proceeds in the presence of solar energy, which supplies the necessary energy required to drive the reaction process [11]. The solar energy plays an important role since much of the natural purification of aqueous systems including lagoons, ponds, streams, rivers and lakes is effected by sunlight, which initiates the breakdown of organic molecules into simpler molecules, and ultimately to carbon dioxide and other mineral products [12].

Tin chalcogenides are semiconductor materials that have found diverse application in photocatalysis, solar cells, Li-ion batteries, switches, light emitting diodes, gas sensors and holographic recording mediums [13]. Generally, they show intense absorption across the electromagnetic spectrum, with narrow band gaps [14]. Other advantages of these tin based compounds include their relative abundance, and less toxicity compared to most metals used as semiconducting materials. They also have tunable band gaps and controllable morphologies [14]. Thus, there has been growing interest in the synthesis of tin chalcogenides. SnS is a n-type semiconductor with a band gap of 2.18–2.44 eV, and interesting electrical and optical properties [15]. It has a CdI_2_-related crystal structure, which consist of two layers of hexagonally closed packed sulfur anions with sandwiched tin cations, which are octahedrally coordinated to the closest six neighboring sulfur atoms [16]. Similarly, SnO_2_ is a n-type semiconductor. It has been used in diverse electrochemical and catalytic applications including solar cells, transparent coating materials, heat mirrors, gas sensing and water treatment due to its unique properties [17,18,19,20,21,22]. It has a high excitonic bonding energy of 130 eV with a direct band gap energy of 3.7 eV [22]. SnO_2_ has attracted much attention due to the exhibition of some catalytic properties and novel properties such as the quantum size effect on photochemistry and nonlinear optical properties [23,24]. The control of the morphology of SnO_2_ is of great importance due its interesting size- and shape-dependent properties [24].

Different approaches have been employed for the syntheses of these chalcogenides with diverse morphological variations. A variety of methods, such as gas phase, laser ablation, sol-gel, solvothermal, hydrothermal, mechanochemical, and pyrolysis of precursor compounds, have been reported [24,25,26]. The properties and performances of nanostructured materials are closely related to size, morphology, crystallinity, crystal defect and surface property [26]. These properties could also be influenced by the method and conditions of preparations [26]. Although several reports exist on the synthesis of SnO_2_ and SnS_2_ nanoparticles, studies involving the use of a single precursor compound for both chalcogenides, without the introduction of any other material, are very rare. The thermal decomposition of a single-source precursor such as dithiocarbamates [27,28], diselenocarbamates [29], semi-/thiosemicarbazone [27,30], carboxylates [31,32] and alkoxides [33] complexes has proven to be a very important route in the synthesis of metal chalcogenides [34]. The decomposition process of dithiocarbamate complexes often proceeds via a thiocyanate intermediate, under inert conditions, which then decomposes to give corresponding metal sulfides as the final residue [35]. Organotin(IV) dithiocarbamate complexes have shown great potential as single-source precursors for the synthesis of clean tin sulfide (of different phases) or oxides nanoparticles, depending on the reaction conditions (inert and in air respectively) [36,37].

In this study, we herein report the synthesis and photocatalytic degradation of methylene blue dye using tin chalcogenides: SnS_2_ and SnO_2_. Both compounds were prepared from diphenyltin(IV) p-methylphenyldithiocarbamate complex via the solvothermal method (under inert condition) and direct pyrolysis (in air). The morphological and optical properties of these particles were also investigated using X-ray diffraction (XRD) technique, scanning electron microscope (SEM) and Ultraviolet-visible (UV-vs) spectroscopy. Methylene blue (MB), a common organic pollutant (dye) in most waste water [2], was used in this study as a model pollutant to investigate the photocatalytic potency of these compounds.

## 2. Materials and Methods

Chemicals used in this research were purchased from Merck chemicals (Darmstadt, Germany) and utilized without purification. The prepared complex was analyzed using nuclear magnetic resonance spectrophotometer (Bruker Avance III 600 MHz) (^1^H, ^13^C and ^119^Sn NMR) (Billerica, MA, USA), while the infrared spectrum was obtained on a Bruker Alpha-P FTIR spectrophotometer (Billerica, MA, USA). The percentage compositions (C, H, N, and S) of the complexes were analyzed using Elementar, Vario EL Cube (Langenselbold, Germany). Thermogravimetric and differential thermogravimetric analysis (TGA/DTG) of the synthesized compound was achieved in a SDTQ 600 Thermal analyzer (Newcastle, DE, USA). Furthermore, the phases of the obtained nanoparticles were identified using X-ray diffraction (XRD) measurements (at a scanning rate of 0.0018 ^o^/min, using a Rőntgen PW3040/60 X’Pert Pro XRD diffractometer equipped with nickel filtered Cu Ka radiation (k = 1.5418 Å) at room temperature) (Shanghai, China). The morphology of these nanoparticles was studied using a TECNAI G2 (ACI) transmission electron microscopy (TEM) (Hillsboro, OR, USA) with an accelerating voltage of 200 kV. The optical property study was achieved using ultraviolet–visible spectrophotometer (UV-1901 Agilent Technology, Cary series UV–vis spectrometer, (Santa Clara, CA, USA). Pyrolysis of the precursor compound was carried out at 400 °C (air) in a muffle furnace (Muffle furnace L 3/12, Nabertherm GmbH, Bahnhofstr, Germany).

### 2.1. Synthesis of Sodium p-Methylphenyldithiocarbamate (NaL)

The preparation of the ligand followed an already reported procedure with some modifications such as the use of NaOH instead of KOH [38].

### 2.2. Synthesis of the Diphenyltin(IV) p-Methylphenyldithiocarbamate Complex [(C_6_H_5_)_2_SnL_2_]

Diphenyltin(IV) chloride (0.005 mol) in 10 mL of cold ethanol (4 °C) was added to some freshly prepared sodium salt of *p*-methylphenyldithiocarbamate in ethanol solution. The obtained mixture was then stirred at 4 °C for about 2 h to give white precipitates. The white precipitated product was washed with excess ethanol, filtered, and dried under vacuum for the whole day.

**[(C_6_H_5_)_2_Sn(L)_2_]**: Yield: 2.82 g (74.80%); M.pt.: 192–194 °C; Selected FTIR, υ (cm^−1^): 1508 (C=N), 1247 (C_2_–N), 998 (C=S), 2949 (–CH), 3056 (=CH), 3145 (N–H) 531 (Sn–C), 371 (Sn–S); ^1^H NMR (DMSO) δ (ppm) = 7.48–7.20 (m, 8H, N–C_6_H_4_–CH_3_–), 2.36 (s, 6H, Ar–CH_3_), 5.29 (s, 2H, N–H), 7.58–7.49 (m, 10H, Sn–C_6_H_5_); ^13^C NMR (DMSO) δ (ppm) = 200.01 (–NCS_2_), 135.46, 130.20, 129.87, 125.51 (N–C_6_H_4_–CH_3_), 21.06 (Ar–CH_3_), 140, 135.61, 130.11, 128.74 (Sn–C_6_H_5_); ^119^Sn NMR (CDCl_3_): δ ppm = −315.96;

C_28_H_26_N_2_S_4_Sn (637.5): C, 52.75; H, 4.11; N, 4.39; S, 20.12; Found: C, 52.25; H, 4.29; N, 4.01; S, 19.99.

### 2.3. Synthesis of Tin Disulfide Nanoparticles (SnS_2_)

The synthesis of SnS_2_ followed a similar report from our research group, with some modifications [37]. In the heat-up approach, 1 g of **[(C_6_H_5_)_2_SnL_2_]** was dispersed into a 20 mL of oleylamine in a 200 mL two necked round bottom flask. The mixture was degassed while being stirred for 10 min before heating up to 120 °C under N_2_ gas. The stirring mixture was held for 20 min and then heated up and maintained at 170 °C. After 1 h, the obtained mixture was kept for a few minutes and allowed to cool to 70 °C. This was followed by the addition of excess methanol for the precipitation of the nanoparticles. The obtained precipitate was washed and centrifuged several times to obtain the purified nanoparticles.

### 2.4. Synthesis of Tin Dioxide Nanoparticles (SnO_2_)

Similarly, to a reported literature procedure [39], about 2.5 g of the precursor complex **[(C_6_H_5_)_2_SnL_2_]** in a crucible was placed in the furnace and heated at 400 °C for 2 h. After the pyrolysis, the residue left in the crucible was cooled to room temperature and then collected for analysis.

### 2.5. Evaluation of the Photocatalytic Activities of the Nanoparticles

The photocatalytic properties of the as-synthesized nanoparticles were evaluated by their degradation efficiency against methylene blue (MB) under a UV–visible light irradiation (k > 300 nm), at room temperature. The photocatalytic potentials of these materials were carried out in a photo-reactor equipped with a 160 W high pressure mercury lamp. In a typical procedure [40], an aqueous solution of methylene blue (MB) (100 mg/L) was prepared by dissolving 0.10 g of methylene blue in 1 L deionized water. Then, 10 mg of the as-synthesized nanoparticles was introduced into 50 mL solution of dye and stirred magnetically for 2 h to establish equilibrium in the dark. The resulting suspension was then stirred at a regular speed while being irradiated in the photo-reactor. Aliquots of about 3 mL at different intervals were taken and the absorption spectra of these aliquots were obtained [41].

## 3. Results

### 3.1. Synthesis of the Ligand (L) and Complex [(C_6_H_5_)_2_SnL_2_]

**Scheme 1:**
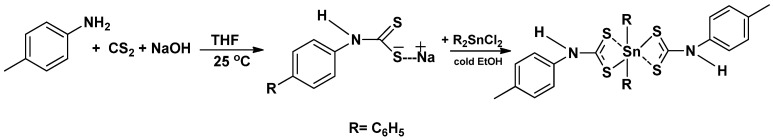
Synthesis route to diphenyltin(IV) and *p*-methylphenyldithiocarbamate **[(C_6_H_5_)_2_Sn(L)_2_].**

Dithiocarbamate ligands obtained from primary amines are generally less stable compared to those obtained from secondary amines due to the presence of the acidic hydrogen on the nitrogen [38,42]. The synthesis of dithiocarbamate ligands from primary amine may sometimes be carried out under an inert atmosphere, as in the case of the *p*-methylphenyl dithiocarbamate **L**. This is because of their instability, which often leads to their decomposition into their corresponding isothiocyanate [43]. The complexes were prepared by the reaction of the ligands with the respective organotin salt, as shown in Scheme 1. The reaction proceeded by the replacement of an equivalent number of chloride ions of the organotin(IV) salt by the ligand. The complex was characteristically white, soluble in dichloromethane, chloroform, dimethylsulfoxide and sparingly soluble in alcohols.

### 3.2. Spectroscopic Studies of the Precursor Complex [(C_6_H_5_)_2_Sn(L)_2_]

Fourier transform infra-red spectra of dithiocarbamate ligands and complexes have been widely reported. The FTIR bands of the ligands and complexes were assigned following other similar spectroscopic reports [42]. A stretching vibration band due to the partial double bond character of C–N was observed at 1508 cm^−1^ in the spectrum of the complex. Also, the presence of a strong band at 998 cm^−1^, ascribed to C–S stretching vibration, suggested a bidentate coordination between the diphenyltin(IV) moiety and the dithiocarbamate ligand [44]. Furthermore, a low intensity peak at 371 cm^−1^ was also observed, which was ascribed to the presence of the Sn–S bond [45,46].

**Figure 1:**
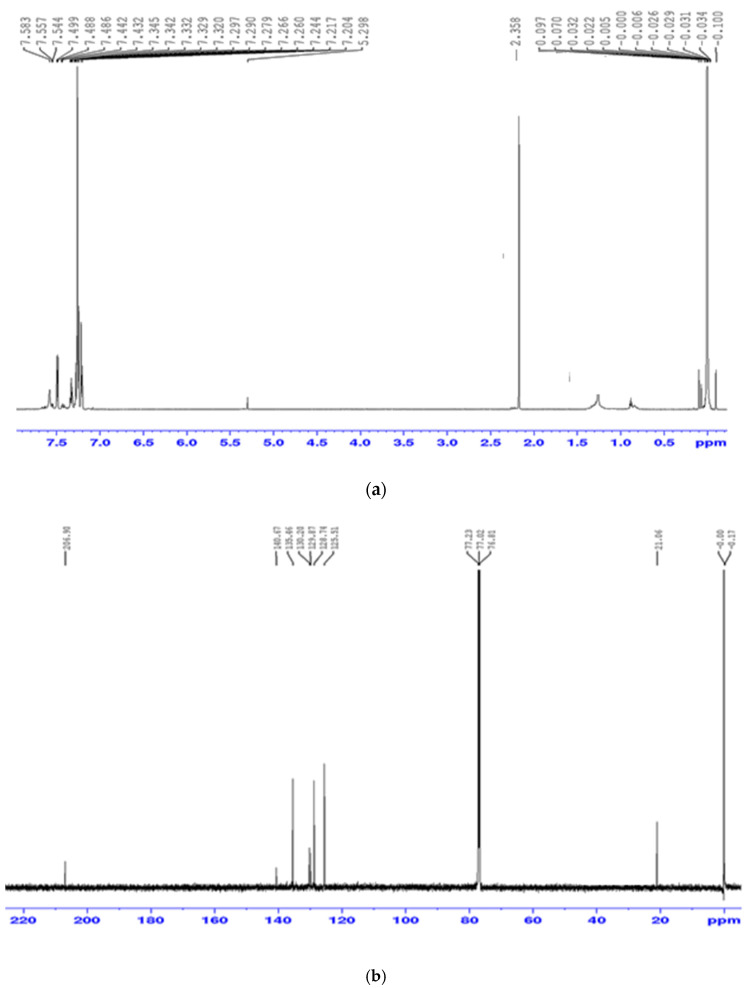
^1^H (**a**) and ^13^C (**b**) spectra of diphenyltin(IV) *p*-methylphenyldithiocarbamate **[(C_6_H_5_)_2_Sn(L)_2_]**.

In the ^1^H NMR spectrum, the protons of the aromatic group appeared as a multiplet, in the downfield region between 7.47 and 7.12 ppm, as shown in Figure 1a. The peaks with the chemical shift in this region were attributed to the proton signals that are ortho to the carbon of the thioureide group because they are more deshielded due to the electronegative N atom and the proximity to the –CS_2_ group [38]. Similarly, the signals due to the phenyl groups on the organotin moiety were found as a complex multiplet in the same range as the diphenyltin ring of the dithiocarbamate moiety. The signal due to the protons of the methyl group found on the para position of the ring in the complex appeared at 2.36 ppm. The signal observed at a higher frequency in the complex around 5.29 ppm has been ascribed to the proton of the N-H, and its position is slightly higher because of the electronegative N atom [47,48].

The ^13^C NMR spectrum (presented in Figure 1b) showed a weak signal at 207 ppm, ascribed to the thioureide carbon atom (–NCS_2_). The region in which these peaks occurred in the complex suggested the contribution of the double bond character of the N–C bond in the dithiocarbamate moiety [38]. The aromatic carbon and the diphenyltin carbon signals resonated within the same range of 140–125 ppm [47]. Furthermore, the signals ascribed to the para-methyl carbon of the complex resonated at about 21 ppm [38]. The ^119^Sn NMR spectrum of the complex showed a peak at approximately −315 ppm, which was suggestive of a hexa-coordinated geometry around the Sn metal.

### 3.3. Thermogravimetric Analysis (TGA) of Diphenyltin(IV) and p-Methylphenyldithiocarbamate [(C_6_H_5_)_2_SnL_2_]

**Figure 2:**
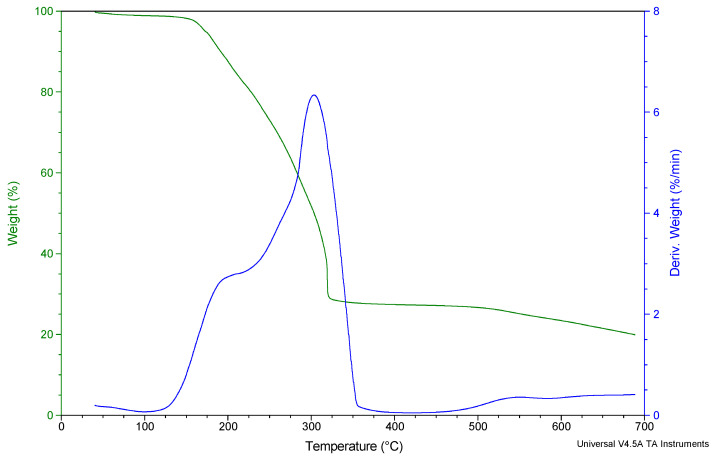
Thermogravimetric and differential thermogravimetric (TG/DTG) curves of diphenyltin(IV) *p*-methylphenyldithiocarbamate obtained under nitrogen atmosphere (75 mL/min), heating rate 10 °C/min.

The thermogravimetric and the differential thermogravimetric (TG/DTG) plots of the complex show a two-step decomposition pathway, as shown in Figure 2. The data obtained from the TG/DTG plots are summarized in Table 1. The first step occurred in the temperature range of 100–217 °C. The mass found after this stage was 82.98% of the starting mass and this could be attributed to the loss of CH_3_–C_6_H_4_ from the ligand molecule of the complex, and agrees well with the calculated value (calc. 83.21%). This was followed by a second and final decomposition in the range 230–321 °C to give a black residue. The mass found was 50.10% of the starting mass and this agreed well with the calculated value of Sn_2_S_3_ (calc. 52.10%) [49]. The observed tin sulfide phase obtained indicates that, as the temperature goes higher than 200 °C, different phases of tin sulfide are obtainable, which implies that the phase of the residue is temperature dependent. Hence, to obtain the desired tin sulfide (nanoparticle) phase, the thermolysis under nitrogen was carried out at 170 °C.

### 3.4. X-ray Diffraction Study of the Synthesized SnS_2_ and SnO_2_ Nanoparticles

**Figure 3:**
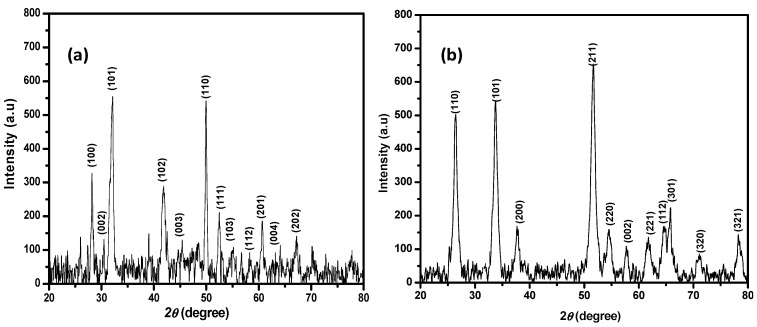
The obtained XRD pattern of (**a**) SnS_2_ and (**b**) SnO_2_ synthesized from diphenyltin(IV) *p*-methylphenyldithiocarbamate.

The XRD patterns of the synthesized nanoparticles (SnS_2_ and SnO_2_) are presented in Figure 3a,b. The observed diffraction peaks (Figure 3a) at 2θ = 28, 30, 32, 42, 46, 50, 51, 55, 58, 60, 63, 67 and 70 were indexed as (100), (002), (101), (102), (003), (110), (111), (103), (200), (201), (004), (202) and (113) diffractions, respectively. These were found to match with the hexagonal phase of SnS_2_ nanoparticles, with JCP2 card No. 40–1467 (lattice parameters a = 3.648 Å, c = 5.898 Å). The sharpness of these peaks indicated good crystallinity, while the absence of any other peak, such as SnO_2_, SnS and Sn, suggests that pure phase SnS_2_ nanoparticles was obtained [36]. Furthermore, the preferred orientation of the synthesized SnS_2_ nanoparticles was towards the (101) plane, similar to the earlier report for SnS_2_ [50]. The diffraction pattern obtained from the calcined complex at 400 °C confirmed the formation of SnO_2_ nanoparticles. These SnO_2_ nanoparticles possess a tetragonal structure, with a JCP2 card No: 41–1445 (lattice parameters a = 4.738 Å, c = 3.187 Å) [51]. The XRD spectra, shown in Figure 3b, indicate that the peaks are somewhat broader than those observed for the SnS_2_ nanoparticles, suggesting a smaller crystallite diameter [52]. The obtained diffraction pattern for SnO_2_ nanoparticles suggests that the preferred growth orientation is in the direction of the (211) plane, similar to those reported in the literature [22,53]. In addition, the average crystallite size, estimated from the 101 peak of the XRD data using Scherrer’s equation [54], indicated estimated particle sizes of 68.8 and 17.62 nm for SnS_2_ and SnO_2_ nanoparticles, respectively.

### 3.5. Morphology of the Synthesize SnS_2_ and SnO_2_

**Figure 4:**
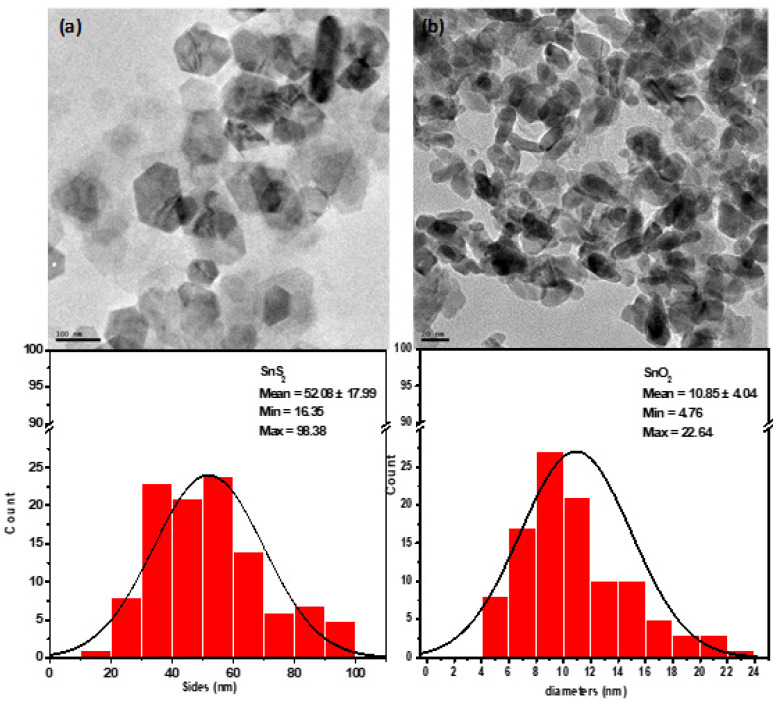
Transmission electron microscopy (TEM) images and histogram distribution plot of (**a**) SnS_2_ (sides) and (**b**) SnO_2_ (diameters) nanoparticles.

The morphology and size of the obtained nanoparticles were studied using transmission electron microscope (TEM). Figure 4a,b shows the morphologies of both SnS_2_ and SnO_2_ nanoparticles in different magnifications. An irregular array of hexagonal plate was observed for the SnS_2_ (Figure 4a) with an average side of 52.08 ± 17.99 nm. This observed shape is similar to those reported by Wang et al. [55] and Li et al. [56], which displayed a relatively better uniform structure. Furthermore, Mali et al. [57] also reported similar features, which suggested that the (101) and (110) preferred orientations in the XRD patterns might have influenced the formation of hexagonal sheets of SnS_2_ [57]. The morphology of the SnO_2_ nanoparticles was completely different from what was observed for SnS_2_, as somewhat spherical nanoparticles which tended towards a short rod were obtained [54]. These particles were also smaller than the SnS_2_ nanoparticles. The average particle diameter was found to be 10.85 ± 4.043 nm for SnO_2_, which was within the estimated size obtained from the XRD.

### 3.6. Ultraviolet-Visible Absorption Spectra

**Figure 5:**
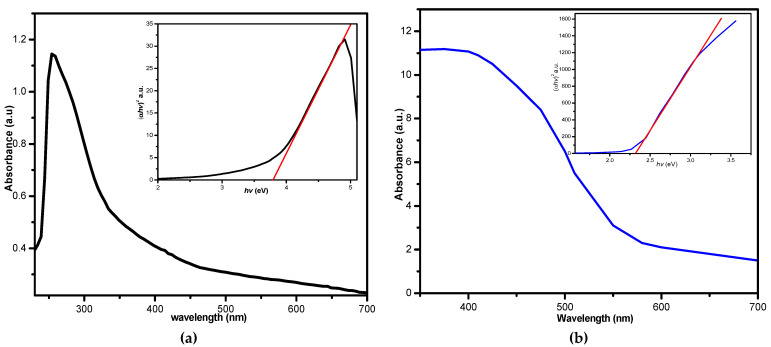
UV-vis spectra and Tauc’s plots (inset) of (**a**) SnO_2_ and (**b**) SnS_2_ nanoparticles.

Semiconductors are known for their good optical properties, and hence found usage in optoelectronic materials [58]. The optical properties of the as-synthesized materials were studied using UV-vis spectroscopy and the obtained spectra are presented in Figure 5a,b for the SnO_2_ and SnS_2_ nanoparticles, respectively. Their band gap energies (eV) were also estimated using the theory of optical absorption for direct band gap semiconductors [13], and Tauc’s plots for both materials are presented as an inset in Figure 5a,b. TheSnS_2_ nanoparticles showed a broad absorption around 405 nm, while the SnO_2_ nanoparticles exhibited a strong absorption at 254 nm, with band gap energies of 2.31 and 3.79 eV respectively. These observed band gap energies were found to be within the range of those reported in the literature [13,58,59,60,61,62].

### 3.7. Photocatalytic Study

**Figure 6:**
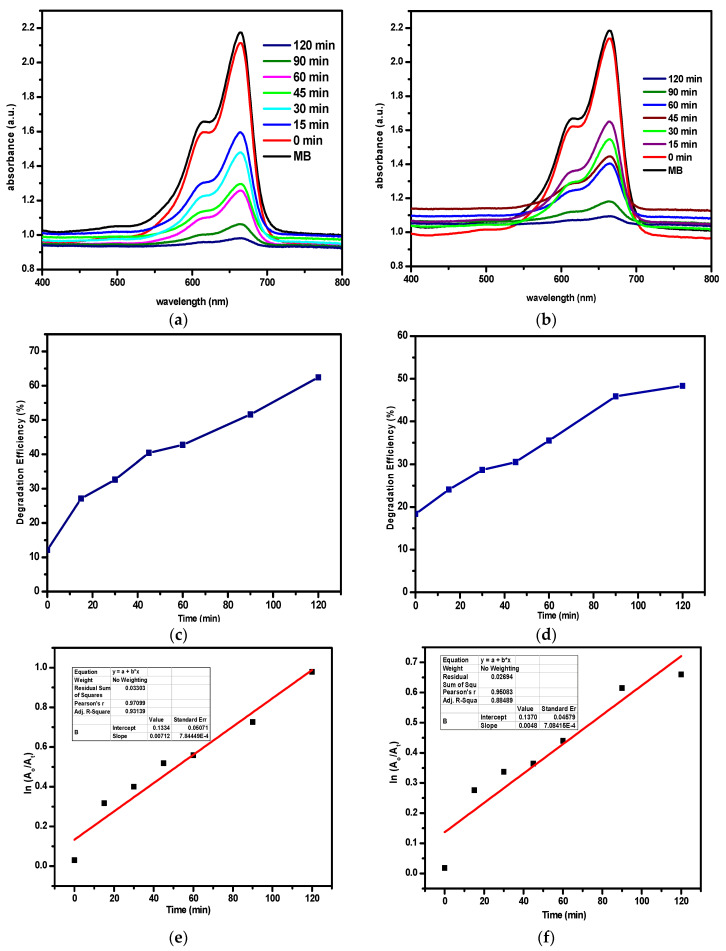
UV–vis spectra of (**a**) SnS_2_ and (**b**) SnO_2_ nanoparticles regarding the photocatalytic degradation of MB; degradation efficiency in percentages of (**c**) SnS_2_ (**d**) SnO_2_; plot of ln A_o_/A_t_ against time showing the first order kinetics of (**e**) SnS_2_ (**f**) SnO_2_.

**Figure 7:**
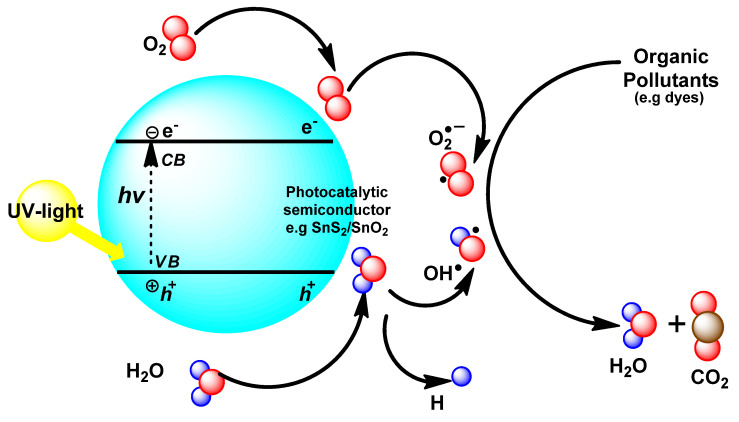
Schematic representation of the mechanism of degradation of organic pollutants through the irradiation of semiconductor and excitation of electrons in the valence band.

The photocatalytic activities of the nanoparticles were evaluated with the aid of a UV-spectroscopy and the obtained absorption spectra for the degraded dye are presented in Figure 6a,b for both SnS_2_ and SnO_2_ nanoparticles, respectively. The absorption maxima for MB is around 665 nm (see Figure 7) due to the presence of the π-system within the dye molecules [63]. The photodegradation efficiency of these materials was estimated using the formula:

(1) $$\mathrm{Degradation}\;\mathrm{Efficiency}\;(\%)=\frac{A_{0}-A_{t}}{A_{0}}\;\times \;100\%$$

As shown in the figures, the type of nanomaterial used affects the degradation efficiency of MB under the UV-visible light irradiation. The as-synthesized SnO_2_ nanoparticles exhibited a degradation efficiency of 48.33% after 120 min reaction, while the SnS_2_ nanoparticles showed an efficiency of 62.42% after the same duration of time. Furthermore, the plot of ln(*A_o_*/*A_t_*) against irradiation time presented in Figure 6c,d shows a linear correlation, suggesting a pseudo first-order kinetics. The obtained rate constant (k) reflects a good absorption rate [40]. The rate constant and correlation coefficient are suggestive that the SnS_2_ nanoparticles have a better degradation potential than the SnO_2_ nanoparticles. The observed differences in their photocatalytic activities may be due to a combination of several factors such as the nature of the nanoparticles, band gap, morphology, crystal defect and photochemical stability [15]. Generally, the mechanism of heterogeneous photocatalysis for the degradation of organic pollutants involves the absorption of sufficient energy from light by the photocatalytic semiconductor such as SnS_2_/SnO_2_ nanoparticles, as presented in Figure 7. The absorbed light energy causes the excitation of electrons from the valence band (VB) of the semiconductor photocatalyst into the conduction band (CB). This process leads to the formation of a reactive electron–hole pair, which then migrates to the semiconductor–water interface to participate in redox reactions with the surrounding species. The outcome of this interaction consequently leads to the degradation of the pollutants in the medium [64]. In order to maximize the absorption of the solar radiation, it is important for the semiconductor to have a band gap energy within the solar spectrum, because the narrowness of the band gaps has been reported to play a vital role in the amount of photons it could absorb at a given time. Hence, in this case, the SnS_2_ nanoparticle with a narrower band gap energy showed better efficiency when compared to its SnO_2_ counterpart.

Dyes naturally undergo degradation in air and under sunlight [65,66]. However, this degradation process is usually slow; hence, the introduction of a semiconductor photocatalyst to speed up the process is a significant process. The use of light as a source of energy plays a key role in the photocatalytic process. Related studies have shown that these semiconductors exhibited no appreciable catalytic decomposition of dye molecules in the dark phase, often used as a control experiment [7,67]. These studies confirmed that photocatalytic reactions rarely proceed in the absence of light, even in different organic dyes and semiconductor materials [7].

## 4. Conclusions

A new diphenyltin (IV) complex of dithiocarbamate derived from a primary amine was successfully synthesized and characterized. Spectroscopic analyses suggested that the *p*-methylphenyldithiocarbamate ligand was coordinated in a bidentate fashion to the central tin atom which was bonded to the biphenyl groups. The potential of the complex as a good precursor compound for the synthesis of SnS_2_ and SnO_2_ was established. The obtained nanoparticles were optically and structurally characterized. Their morphologies showed that hexagonal shaped sheets were obtained for SnS_2_, while the SnO_2_ nanoparticles displayed spherical shapes that tend toward short rods. The optical study showed that both SnS_2_ and SnO_2_ gave a direct band gap of 2.31 and 3.79 eV, respectively. The photocatalytic evaluation of both compounds, using MB as a model pollutant, showed that SnS_2_ exhibited better degradation efficiency compared to SnO_2_ nanoparticles under similar conditions. This novel complex has shown the capacity as a useful precursor complex for the synthesis of useful tin chalcogens under varying conditions.

## Figures and Tables

**Table 1:** Thermal analysis data of diphenyltin(IV) and *p*-methylphenyldithiocarbamate.

Temperature Range of Decomposition (°C)		DTG Peak T (°C)		Product Obtained after Decomposition		Mass of Residue (mg) Found (Calc)	
1st step	2nd step	1st step	2nd step	1st step	2nd step	1st step	2nd step
100–217	230–321	216	303	(CH_3_-Ph) (HNCS_2_)_2_ Sn (Ph)_2_	Sn_2_S_3_	11.90 (11.93)	7.19 (7.47)
